# Increased circulation of human adenovirus in 2023: an investigation of the circulating genotypes, upper respiratory viral loads, and hospital admissions in a large academic medical center

**DOI:** 10.1128/jcm.01237-23

**Published:** 2023-12-19

**Authors:** Omar Abdullah, Amary Fall, Eili Klein, Heba H. Mostafa

**Affiliations:** 1 Department of Pathology, Division of Medical Microbiology, Johns Hopkins School of Medicine, Baltimore, Maryland, USA; 2 Department of Emergency Medicine, Johns Hopkins School of Medicine, Baltimore, Maryland, USA; 3 Center for Disease Dynamics, Economics, and Policy, Washington, DC, USA; Mayo Clinic Minnesota, Rochester, Minnesota, USA

**Keywords:** adenovirus, genotyping, HAdV-B3, ddPCR, respiratory infection

## Abstract

An increase in the circulation of human adenoviruses (HAdV) in 2023 was notable. HAdV genotypes circulating were characterized. Viral loads, clinical presentations, and outcomes were associated with the genotypes. Remnant respiratory samples positive for HAdV after standard-of-care testing at the Johns Hopkins Microbiology laboratory (*N* = 270) were collected for genotyping by next-generation sequencing of the hexon gene. HAdV loads in respiratory samples were assessed using droplet digital PCR. The association between predominant genotypes, outcomes, and viral loads was evaluated. Of a total of 249 samples with characterized HAdV genotypes, 179 (71.9%) were genotype B3. HAdV-B3 was associated with a statistically significant increase in viral loads in respiratory samples, specifically in patients 5 years and younger. Patients infected with HAdV-B3 were primarily in the age group 3–5 years in contrast to patients infected with non-B3 genotypes who were younger than 3 years. Strict criteria for defining HAdV-related admission identified a hospitalization rate of 14.8%. Infections with HAdV-B3 were not associated with an increased likelihood of HAdV-related admissions. The circulation of HAdV-B3 in 2023 after at least 2 years of reduced detection likely contributed to the increased number of cases.

**IMPORTANCE:**

The circulation of human adenoviruses (HAdV) increased in 2023. In this manuscript, we show that HAdV-B3 was predominant in 2023 in a cohort characterized by the Johns Hopkins Hospital System. We also show that HAdV-B3 was associated with an increase in viral loads in respiratory samples and provide a correlation with the clinical presentations and outcomes.

## INTRODUCTION

Human adenoviruses (HAdV) belong to seven species within the genus *Masadenovirus*, family *Adenoviridae*. HAdV is associated with a spectrum of diseases that includes respiratory infections, keratoconjunctivitis, and gastroenteritis. Disease associations and different outcomes with specific types have been reported with types 1–5, 7, 14, 21, and 55 frequently associated with upper and lower respiratory tract infections (1) [respiratory HAdV types belong to species B (HAdV-3, 7, 11, 14, and 21), C (HAdV-1, 2, 5, and 6), and E (HAdV-4)] (2). Respiratory disease outbreaks and sporadic infections with HAdV contribute to up to 10% of respiratory tract infections (3, 4).

HAdV genomic surveillance in the United States is voluntary and infections with HAdV are not notifiable. The most commonly reported types in the US between 2003 and 2016 were 1, 2, 3, 4, 7, and 14, with a predominance of type B3 in 2015 and 2016 (5). Type B3 was also predominant between 2017 and 2019 in a study from China that characterized HAdV infections of hospitalized patients with severe respiratory disease (3) and in another study that characterized samples collected in 2014 (6). Species B and C dominated in a study from Argentina that characterized the circulating types in the period between 2000 and 2018 (7). Our genomic surveillance of HAdV types from December 2020 to April 2022 showed a predominance of C1 (49.5%) and C2 (34.3%) types and no detection of HAdV-B3 (8).

A notable increase in the circulation of HAdV at the Johns Hopkins Hospital System in 2023 was consistent with a national increase (9) and included laboratory positivity rates and hospital encounters. In this study, we characterized the HAdV types associated with respiratory tract infections in the time frame from January to June 2023 and correlated HAdV types with the clinical presentations, outcomes, and viral loads in the respiratory samples.

## MATERIALS AND METHODS

### Ethics and study samples

The research was conducted with a waiver of consent (Johns Hopkins IRB protocol IRB00247284). Remnant respiratory specimens positive for HAdV [standard of care testing is performed with the ePlex respiratory pathogen panels (10)] were collected for the study (convenience sample, all available left-over specimens were enrolled in the study).

### Study cohort

A total of 349 HAdV-positive respiratory samples (nasopharyngeal swabs) were tested by the Johns Hopkins Microbiology laboratory between 2 January and 4 June 2023. A total of 270 [268 nasopharyngeal swabs and 2 bronchoalveolar lavage (BAL)] remnant specimens (all samples were from unique patients) were retrieved for typing, viral load quantification, and clinical data analysis (Table S1). Notably, the 2 BAL samples were excluded from the viral load analyses to restrict comparisons to the same respiratory compartment.

### Clinical data

Clinical data were extracted in bulk from the electronic medical record system (EHR). A conservative approach was used to determine HAdV-related admissions, which included presence of viral-related symptoms, a positive finding of HAdV within 48 h of admission, and a negative result for all other tested pathogens. The 12 patients that were “excluded” (Table 1), had a positive HAdV result collected 48 h or more after hospital admission. The Johns Hopkins laboratory serves the whole Johns Hopkins Hospital System which includes two academic hospitals, three community hospitals, and outpatient centers and altogether covers a large geographical area in the State of Maryland, Virginia, and DC. All hospitals and outpatient centers utilize the same integrated EHR.

**TABLE 1:** Demographics of patients used in the study and the characterized HAdV types^*c*^

	HAdV-related admission	Non-related admission	Not-admitted	Excluded
N	40	11	207	12
Female	20 (50.0%)	4 (36.4%)	97 (46.9%)	6 (50.0%)
Mean patient age (SD)	18.1 (25.2)	12.0 (22.7)	5.6 (7.4)	21.5 (31.6)
Age categories				
0–2	9 (22.5%)	4 (36.4%)	68 (32.9%)	8 (66.7%)
3–5	12 (30.0%)	1 (9.1%)	71 (34.3%)	0 (0.0%)
6–17	8 (20.0%)	5 (45.5%)	58 (28.0%)	0 (0.0%)
18+	11 (27.5%)	1 (9.1%)	10 (4.8%)	4 (33.3%)
Race/ethnicity				
Black	16 (40.0%)	5 (45.5%)	53 (25.6%)	3 (25.0%)
Hispanic	2 (5.0%)	2 (18.2%)	57 (27.5%)	2 (16.7%)
Other	5 (12.5%)	3 (27.3%)	45 (21.7%)	3 (25.0%)
White	17 (42.5%)	1 (9.1%)	52 (25.1%)	4 (33.3%)
Comorbidities				
Atrial fibrillation	3 (7.5%)	1 (9.1%)	1 (0.5%)	1 (8.3%)
Cancer	10 (25.0%)	3 (27.3%)	30 (14.5%)	3 (25.0%)
Cerebrovascular disease	8 (20.0%)	0 (0.0%)	7 (3.4%)	4 (33.3%)
Coronary artery disease	7 (17.5%)	1 (9.1%)	5 (2.4%)	4 (33.3%)
Diabetes	5 (12.5%)	0 (0.0%)	3 (1.4%)	4 (33.3%)
Heart failure	4 (10.0%)	1 (9.1%)	4 (1.9%)	2 (16.7%)
Hypertension	13 (32.5%)	3 (27.3%)	15 (7.2%)	5 (41.7%)
Immunosuppression	24 (60.0%)	6 (54.5%)	26 (12.6%)	10 (83.3%)
Kidney disease	13 (32.5%)	3 (27.3%)	10 (4.8%)	5 (41.7%)
Lung disease^*b*^	15 (37.5%)	6 (54.5%)	38 (18.4%)	4 (33.3%)
Smoker	6 (15.0%)	1 (9.1%)	3 (1.4%)	2 (16.7%)
Outcome				
ICU admission	10 (25.0%)	5 (45.5%)	1 (0.5%)	7 (58.3%)
Supplemental oxygen	22 (55.0%)	6 (54.5%)	6 (2.9%)	11 (91.7%)
Viral load. Log copies/uL (mean/SD)^*a*^	2.0 (2.0)	1.1 (1.8)	3.3 (1.7)	1.9 (1.5)
Coinfections				
Parainfluenza	0 (0.0%)	0 (0.0%)	4 (1.9%)	0 (0.0%)
Coronavirus (non-COVID)	0 (0.0%)	2 (18.2%)	13 (6.3%)	1 (8.3%)
Rhino/entero	0 (0.0%)	9 (81.8%)	51 (24.6%)	2 (16.7%)
RSV	0 (0.0%)	0 (0.0%)	1 (0.5%)	1 (8.3%)
HAdV genotype				
A31	−	−	−	1 (8.3%)
B21	3 (7.5%)	−	−	−
B3	22 (55.0%)	3 (27.3%)	152 (73.4%)	2 (16.7%)
B5	−	−	2 (1.0%)	1 (8.3%)
B7	−	−	1 (0.5%)	−
C1	1 (2.5%)	1 (9.1%)	17 (8.2%)	2 (16.7%)
C2	4 (10.0%)	4 (36.4%)	26 (12.6%)	2 (16.7%)
C5	−	−	1 (0.5%)	−
E4	2 (5.0%)	−	2 (1.0%)	−
Missing	8 (20.0%)	3 (27.3%)	6 (2.9%)	4 (33.3%)

^*a*^ Missing data for 3 HAdV-related admission groups, 1 from the non-related admission group, 5 from the non-admitted group, and 4 from the excluded group.

^*b*^ Lung disease includes sarcoidosis of the lung, chronic obstructive pulmonary disease, asthma, and interstitial lung disease.

^*c*^ Excluded patients refer to hospital-admitted patients who tested positive for HAdV 48 h or more after admission.

### HAdV droplet digital PCR (ddPCR)

Protocol and primer sequences were previously detailed (8). Samples were extracted via the chemagic Viral RNA/DNA Kit following the manufacturer’s instructions (300 µL extracted volume and 60 µL elution volume). The One-Step Kit for Probes was used for ddPCR. The master mix was composed of ddPCR Supermix (5.5 µL), reverse transcriptase (2.2 µL), DTT (1.1 µL), forward primer (0.9 µL of 10 µM), reverse primer (0.9 µL of 10 µM), Probe (0.45 µL of 10 µM FAM), dH_2_O (5.95 µL) for a total of 17 µL per sample. Five microliters of sample eluate was added for a reaction volume of 22 µL. The ddPCR plate was shaken at 3,000 rpm for 1 min, and centrifuged at 1,000 rpm for 10 s. The sample plate and a new plate were loaded onto the droplet generator. The new plate with the generated droplets was loaded onto the Bio-Rad C1000 Touch thermocycler with cycling conditions of hold at 25°C for 3 min, RT at 50°C for 60 min, enzyme deactivation at 95°C for 10 min, 40 cycles of denaturing at 95°C for 3 s and annealing and extension at 55°C for 1 min, then enzyme deactivation at 98°C for 10 min, and hold at 4°C. Droplets were read using the QX200 Droplet Reader and analyzed with the QuantaSoft Analysis Pro 1.0.596.0525 (Bio-Rad). Samples that were too concentrated were diluted at 1:100 and re-tested. Multiple negative control samples were included in each plate and a subset of samples were tested in replicates to exclude cross contamination and ensure reproducibility of the data.

### HAdV hexon amplification and DNA sequencing

The hexon gene sequences of the samples screened positive for HAdV were obtained by nested PCR amplification as described previously (8, 11). Briefly, for the first PCR , the master mix is composed of dH_2_O (33.75 µL), 10× PCR buffer (5.0 µL), dNTP (4.0 µL at 200 µM), Forward Primer 1 (1.0 µL at 10 µM), Reverse Primer 1 (1.0 µL at 10 µM), and Taq polymerase (Roche Diagnostics) (0.25 µL) for a total of 45 µL per sample. Five microliters of sample eluate is then added for a total of 50 µL. Cycling conditions include holding 2 min at 94°C, 35 cycles of 94°C (1 min denature), 45°C (1 min annealing), and 72°C (2 min extension), then 72°C hold for 5 min and a final 4°C hold. The nested PCR master mix is composed of dH_2_O (37.75 µL), 10× PCR buffer (5.0 µL), dNTP (4.0 µL at 200 µM), Forward Primer 2 (1.0 µL at 10 µM), Reverse Primer 2 (1.0 µL at 10 µM), and Taq polymerase (0.25 µL) for a total of 49 µL per sample. One microliter of PCR 1 product is then added for a total of 50 µL. Cycling conditions are the same as for PCR 1. Library prep was performed using the Native barcoding kit (EXP-NBD196) and the NEBNext ARTIC Library Prep Kit according to the manufacturer’s instructions and sequenced using a R9.4.1 flowcells (Oxford Nanopore Technologies) on a GridION (Oxford Nanopore Technologies).

For a subset of samples that failed sequencing (35), the nested PCR was performed as detailed above with the exception of adding 2 µL of PCR 1 product into the PCR 2 master mix. Samples were then sequenced using the P2 Solo (Oxford Nanopore Technologies) and R10 flow cells. This approach helped recover 14 additional genotypes.

The Fastq files generated were analyzed using our in-house pipeline, which comprised several steps. These steps included blasting against a database consisting of all HAdV types reference genomes, selecting the closest reference, running mini_assemble within pomoxis to generate a draft genome, employing minimap2 (12) for alignment, and racon (13) for polishing, using medaka_consensus to further enhance the draft genome and establish a consensus sequence, and finally, evaluating depth with samtools.

### Phylogenetic analysis of HAdV sequences

Alignment was performed using Mafft (v.7.450). Maximum-likelihood trees were developed by IQ-TREE2 (v.2.0.6) with 1,000 bootstrap replicates and visualized with FigTree version 1.4.4. The ModelFinder, implemented in IQ-TREE2, was used to select the best-fitted nucleotide substitution model.

### Statistical analyses

Statistical analyses were performed with non-parametric One-way analysis of variance (ANOVA) or *t* test using the GraphPad Prism 9.5.1 for viral load and age comparisons. The Fisher Exact test was used to compare age groups. Clinical and multivariable logistic regression analyses were performed using STATA/SE 18 to evaluate the odds ratio of HAdV-related admission and the need for supplemental oxygen.

## RESULTS

### Increased detection of HAdV in 2023 at the Johns Hopkins Microbiology laboratory

**Fig 1:**
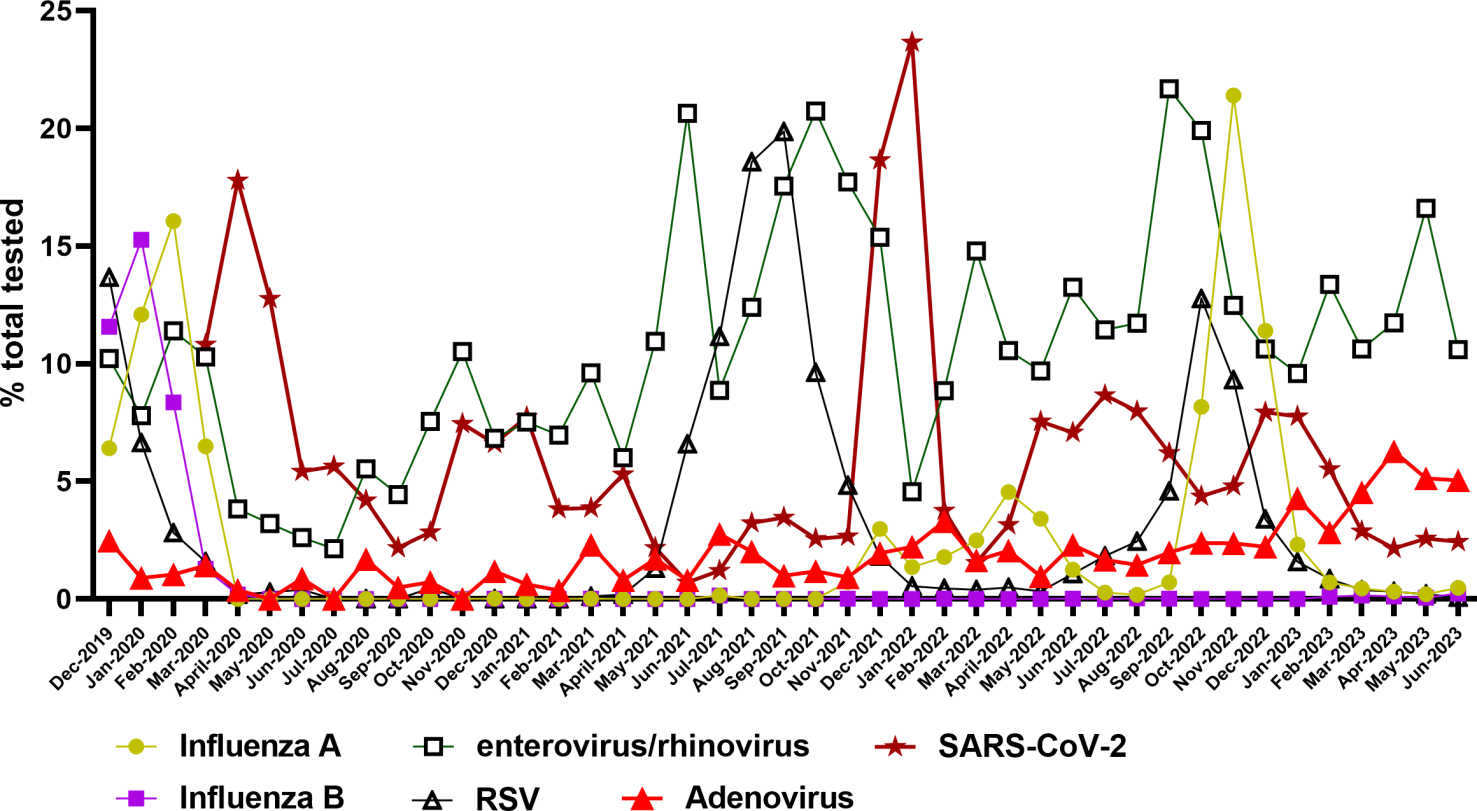
Respiratory virus positivity rates for samples tested across the Johns Hopkins Health System, December 2019–June 2023.

In January 2023, a notable increase in HAdV positivity to 4.3% of all tested upper respiratory specimens was observed (Fig. 1). This marked the highest positivity rate reported since shortly before the COVID-19 pandemic started (Fig. 1). HAdV positivity continued to rise, reaching a peak of 6.3% in April, making HAdV the second most prevalent respiratory virus between April and June 2023, second only to rhinovirus/enterovirus.

Of a total of 349 HAdV-positive samples diagnosed between 1 January and 30 June 2023, 270 samples (from unique patients) were collected for genotyping. The sample collection months included 46 from January, 29 from February, 60 from March, 64 from April, 64 from May, and 7 from June. The majority of the 270 patients were 5 years old or younger (173, 64.1%, Table 1), and the most frequent underlying condition was immunosuppression. A total of 63 patients were admitted (23.3%, Table 1) (HAdV-related admission, non-related admission, and excluded), of them, 40 were classified as HAdV-related. Of the Patients admitted with HAdV infection, 22 (55%) required supplemental oxygen, and 10 (25%) received ICU-level care (Table 1). Notably, viral coinfections were detected in the study cohort in 84 patients (Table 2). The majority of coinfections (62) were with rhinovirus/enterovirus (Table 1).

**TABLE 2:** Clinical characteristics of patients infected with HAdV-B3 and non-B3 types

	B3		All other types	
	179		70	
Age				
All age groups (median, SD)	5	9.7	1	12.03
	N	%	N	%
0–2 years	36	20.1	50	71.4
3–5 years	74	41.3	8	11.4
6–12 years	55	30.7	4	5.7
13–19 years	6	3.4	2	2.9
50 years	5	2.8	4	5.7
>50 years	3	1.7	2	2.9
Gender				
Males	97	54.2	36	51.4
Females	82	45.8	34	48.6
Underlying conditions				
Hypertension	19	10.6	7	10.0
Pregnancy	3	1.7	1	1.4
Lung disease	41	22.9	15	21.4
Kidney disease	14	7.8	6	8.6
Immunosuppression	35	19.6	17	24.3
Diabetes	3	1.7	3	4.3
Heart failure	5	2.8	2	2.9
Atrial fibrillation	2	1.1	0	0.0
Smoker	2	1.1	3	4.3
Cerebrovascular disease	10	5.6	4	5.7
Cancer	31	17.3	6	8.6
Coronary artery disease	7	3.9	3	4.3
Outcome				
HAdV related admission	24	14.0	13	18.6
ICU	7	4.1	4	5.7
Supplemental oxygen	13	7.6	8	11.4
Expired	2	1.2	1	1.4
Chief symptoms				
Total with symptom data	171	95.5	63	90.0
Fever	123	71.9	44	69.8
Emesis	15	8.8	2	3.2
Cough	12	7.0	4	6.3
Abdominal pain	10	5.8	3	4.8
Otitis media diagnosis	11	6.4	6	9.5
Eye infection	6	3.5	2	3.2

### HAdV-B3 is the predominant genotype in 2023

**Fig 2:**
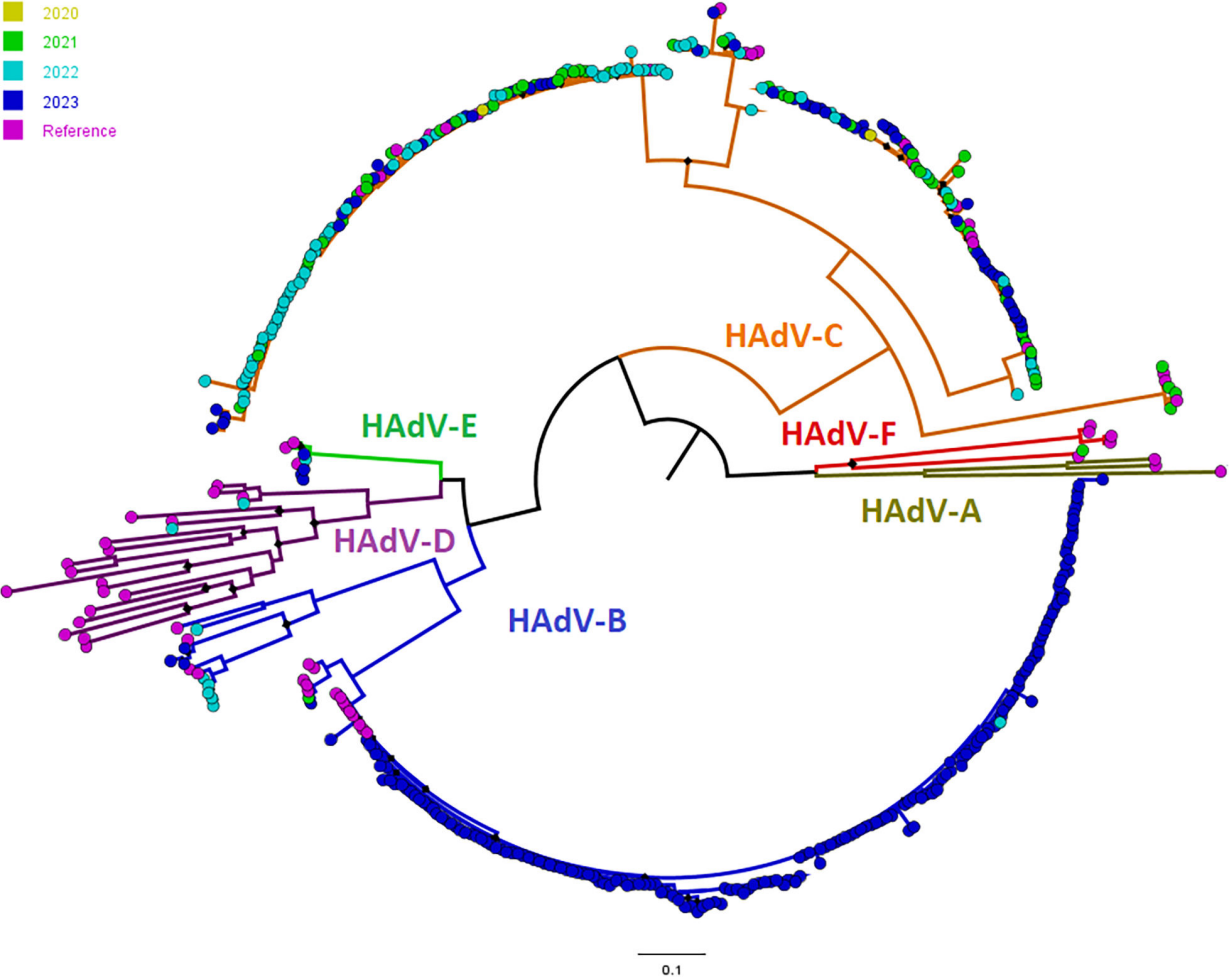
HAdV types characterized at the Johns Hopkins laboratory in 2023 (blue), 2022 (light blue), 2021 (light green), and 2020 (dark yellow). The phylogenetic tree was generated of nucleotide sequences of the hexon gene hypervariable region.

Genotyping of the 270 HAdV positive samples showed that 179 (66.3%) belong to the B3 genotype (Table 1; Table S1, and Fig. 2). Other characterized genotypes included primarily C2 (36, 13.3%) and C1 (21, 7.7%). Genotyping failed for 21 samples (7.8%) (Table 1). Patients infected with the B3 genotype were primarily 3–5 years old (41.3%) followed by children 6–12 years of age (30.7%) (Table 2). These results are in contrast to patients infected with non-B3 genotypes, who were mainly younger than 3 years old (71.4%) (Table 2, Fisher Exact test, *P* < 0.0001). Patients infected with the B3 genotype were less likely to be admitted (14% versus 18.6%) or receive supplemental oxygen (7.6% versus 11.4%) when compared with patients infected with non-B3 genotypes (Table 2) though these differences were not statistically significant (see below). The most frequent symptom for both groups was fever (Table 2). Multivariable logistic regression analysis showed that the age of 18 and older and immunosuppression increased the likelihood of HAdV-related admission and the need for supplemental oxygen (Table 3). Notably, infection with genotype B3 did not increase the likelihood of hospital admission (Table 3).

**TABLE 3:** Multivariable logistic regression

	HAdV -related admission	HAdV -related admission^*a*^	Supplemental oxygen	ICU admission
Female	0.8 (0.3–1.8)	0.7 (0.3–2.0)	0.3 (0.1–0.9)	1.6 (0.5–5.2)
Age categories				
0–2				
3–5	1.2 (0.4–3.7)	1.5 (0.4–6.0)	0.7 (0.2–2.7)	0.5 (0.1–2.4)
6–17	0.7 (0.2–2.4)	0.5 (0.1–2.2)	0.6 (0.2–2.0)	0.9 (0.2–3.9)
18+	9.6 (1.5–61.1)	16.8 (2.0–139.7)	15.1 (1.9–117.6)	0.0 (0.0–6.2)
Race/ethnicity				
Black	1.9 (0.5–6.7)	2.8 (0.6–13.0)	3.2 (0.6–17.1)	2.7 (0.5–15.8)
Hispanic	0.4 (0.1–2.3)	0.3 (0.0–3.6)	2.4 (0.3–16.2)	0.4 (0.0–5.5)
Other				
White	2.7 (0.8–8.9)	3.3 (0.8–13.6)	3.1 (0.6–17.1)	1.4 (0.2–8.9)
Comorbidities				
Atrial fibrillation	0.7 (0.0–17.0)	0.1 (0.0–10.8)	−	2.4 (0.0–347.2)
Cancer	0.5 (0.1–1.8)	0.4 (0.1–2.4)	0.9 (0.2–3.7)	3.2 (0.8–13.3)
Cerebrovascular disease	3.1 (0.5–18.2)	2.7 (0.3–23.2)	4.7 (0.8–27.7)	14.9 (1.5–151.8)
Coronary artery disease	2.2 (0.2–23.2)	1.0 (0.1–17.6)	1.5 (0.1–18.8)	10.6 (0.4–313.4)
Diabetes	2.2 (0.2–19.6)	21.7 (1.6–298.7)	0.1 (0.0–1.0)	1.7 (0.0–122.4)
Heart failure	0.3 (0.0–3.7)	0.3 (0.0–5.5)	5.1 (0.3–79.6)	0.4 (0.0–7.4)
Hypertension	0.5 (0.1–2.4)	0.4 (0.1–2.4)	0.3 (0.0–1.8)	0.0 (0.0–0.6)
Immunosuppression	8.4 (3.1–23.0)	13.3 (4.1–43.3)	4.1 (1.3–13.6)	1.7 (0.4–7.5)
Kidney disease	2.4 (0.5–11.0)	2.4 (0.3–21.1)	7.7 (1.4–43.3)	6.2 (1.0–39.5)
Lung disease	1.1 (0.4–3.0)	1.9 (0.6–5.9)	3.9 (1.4–10.8)	1.4 (0.3–6.0)
Smoker	0.2 (0.0–3.5)	0.3 (0.0–6.9)	0.2 (0.0–2.6)	−
B3		0.9 (0.7–1.2)		
N	258	241^*a*^	258	258

^*a*^ Patients infected with HAdV for which genotyping was unsuccessful were excluded.

### HAdV-B3 respiratory samples have higher HAdV load than other types

**Fig 3:**
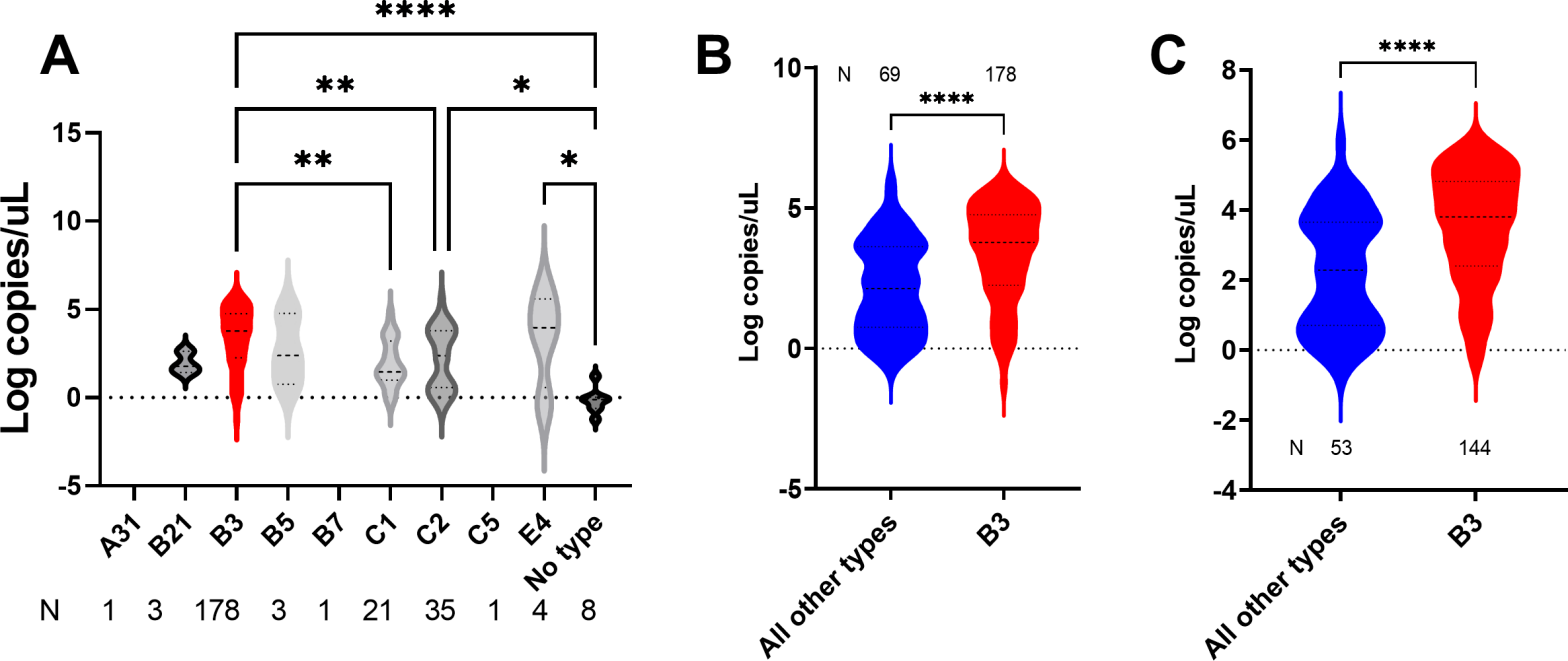
Association of HAdV types with viral loads in respiratory samples for (**A**) genotypes characterized in 2023, (**B**) B3 genotype versus all other characterized types in 2023, and (**C**) B3 genotype versus all other characterized types in 2023 for samples collected within the first week of symptoms. Data shown as violin plots and horizontal lines mark the medians and quartiles. *P < 0.05, **P < 0.01, ****P < 0.0001.

All 270 samples were quantified by ddPCR to determine the association between HAdV genotypes and viral load in respiratory samples. Positive droplets were not detected for a total of 13 samples, which also had failed genotyping (Table S1). In general, lower mean viral load was noted for all samples that did not have a characterized genotype (Fig. 3A). Interestingly, samples with genotype B3 showed the highest average viral loads (3.4 log copies/uL) which was significant when compared to samples with genotypes C1 (1.9 log copies/uL, one-way ANOVA, *P* = 0.007) and C2 (2.2 log copies/uL, one-way ANOVA, *P* = 0.006), (Fig. 3A). B3 samples had a significantly higher mean viral load than all other characterized genotypes (3.4 versus 2.2 log copies/uL, *t* test, *P* < 0.0001, Fig. 3B). Restricting the analysis to specimens collected from patients within the first week of symptoms (Table S1, HAdV-B3, 144 samples, non-B3, 53 samples), a significantly higher mean viral load was still observed with HAdV-B3 samples (3.5 versus 2.2 log copies/uL, *t* test, *P* < 0.0001, Fig. 3C).

### Viral loads and associations with age and disease outcome

**Fig 4:**
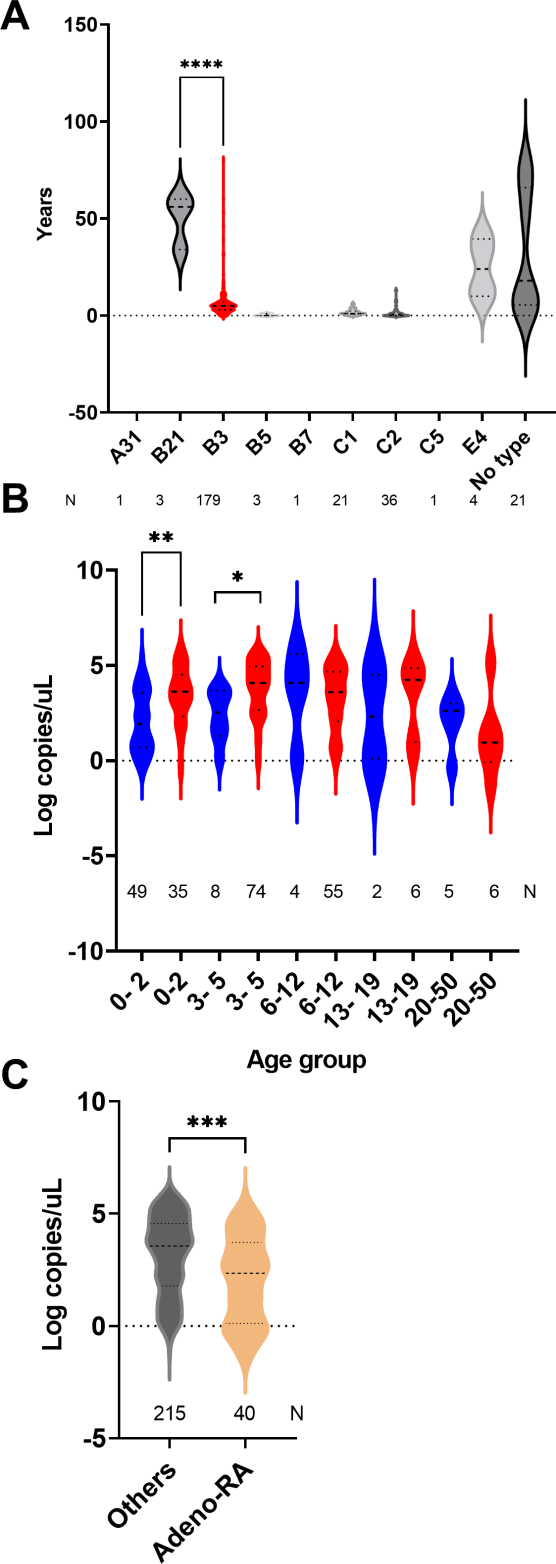
Association of age and disease outcome with HAdV loads in respiratory samples. (**A**) Age of patients in each HAdV genotype group. (**B**) Viral loads per age group in B3 genotype infected (red) versus all other genotypes (blue). (**C**) Viral loads in patients admitted with HAdV infection (Adeno-RA) versus all other HAdV-positive patients. Data shown as violin plots and horizontal lines mark the medians and quartiles. *P < 0.05, **P < 0.01, ****P < 0.0001.

To evaluate if higher viral loads differed by age group, we first compared the median age infected by each genotype. The median ages of patients infected with HAdV-B3, C1, and C2 were 5, 1, and 0.5 years, consecutively (Fig. 4A). Viral loads from samples collected from patients younger than 3 years (3.4 versus 2.1 log copies/uL, *t* test, *P* = 0.0003) or age 3–5 years (3.8 versus 2.4 log copies/uL, *t* test, *P* = 0.02) were higher for the HAdV-B3 groups when compared to other types (Fig. 4B). Samples collected from patients admitted with HAdV infections had lower average viral loads than samples collected from all other groups (2 versus 3.1 log copies/uL, *t* test, *P* = 0.001, Fig. 4C).

## DISCUSSION

Our report demonstrates a predominance of HAdV-B3 during the year 2023, which was associated with an increased circulation of HAdV (9). Additionally, we show that HAdV-B3 infections were associated with a higher HAdV load in respiratory samples compared to non-B3 types. The majority of HAdV-B3 infections were in patients 3–5 years old in contrast to other types that primarily infected patients younger than 3 years. Our data also indicated that, despite the predominance of HAdV-B3, infections of this type were not associated with an increased likelihood of HAdV-related admission. Notably, our cohort exhibited relatively high rates of viral coinfections.

HAdV infections are ubiquitous, and by the age of 10, most children are likely to have been infected with at least one HAdV type. The majority of HAdV-associated respiratory infections occur within the first 5 years of life (1). HAdV genotypes 3, 4, 7, 14, and 21 have been reported to be associated with epidemics of respiratory infections and severe disease (14–17). HAdV-B3 is among the most frequently reported genotypes associated with respiratory infections and epidemic conjunctivitis (18). In our cohort, patients infected with HAdV-B3 were primarily children aged 3–5 years old, and their chief symptoms were fever (71.9%), cough (7%), and otitis media (6.4%). Eye infections were infrequently encountered, reported in only 3.5% of patients infected with HAdV-B3. Admissions and the need for supplemental oxygen were less likely in patients infected with HAdV-B3 when compared to patients infected with other types, an observation that did not reach statistical significance. The age group and percentage of admissions observed in our study for patients infected with HAdV-non-B3 genotypes were consistent with what we reported for HAdV infections in the time frame of 2020–2022 when HAdV-C predominated (8). Older age and immunosuppression were the top two variables associated with increased likelihood of admissions with HAdV.

Although higher viral loads in respiratory samples can be associated with acute infections, when comparing HAdV-B3 samples to non-B3 types collected within the first 7 days of symptoms, HAdV-B3 was associated with higher viral loads. Interestingly, in a prior study, we did not detect any significant differences in viral loads between samples from patients infected with different HAdV genotypes (8). The predominant genotypes in our prior study included HAdV-C1, C2, and C5 (8). The predominance of HAdV-B3 in 2023, after at least two years of reduced circulation, might be related to an increase in the susceptible population, which could explain the increased viral loads, the increased infections in older age groups, and subsequently increased transmission. Prior research found that the hypervariable region of HAdV-B3 circulating strains is diverse and the number of observed variants is notably higher than other HAdV genotypes, which might explain its increased prevalence and faster rate of immune escape (19, 20).

Our study highlights the value of genomic surveillance for understanding patterns of respiratory viral infections and circulation. The limitations of our study include the relatively high rates of viral coinfections which made attributing admissions or disease presentations to HAdV challenging. In addition, differentiating active infection and viral shedding using a molecular diagnostic method is not trivial. Prolonged viral shedding and detection by molecular assays is well documented with multiple respiratory viruses, and can cause confusion in laboratory results’ interpretation and clinical diagnosis (21–31). Moreover, the relatively small size of our cohort limited our outcome analyses and follow-up studies are warranted to validate our results.

## SUPPLEMENTAL MATERIAL

Table S1 jcm.01237-23-s0001.xlsx Cohort of samples and patients used for the study.

ASM does not own the copyrights to Supplemental Material that may be linked to, or accessed through, an article. The authors have granted ASM a non-exclusive, world-wide license to publish the Supplemental Material files. Please contact the corresponding author directly for reuse.
